# A case report of an atypical haemolytic uremic syndrome in pregnancy: something wicked this way comes

**DOI:** 10.1186/s12871-023-02066-4

**Published:** 2023-03-28

**Authors:** Stefano Catarci, Bruno Antonio Zanfini, Mariangela Di Muro, Emanuele Capone, Luciano Frassanito, Maria Teresa Santantonio, Gaetano Draisci

**Affiliations:** Department of Scienze dell’Emergenza, Anestesiologiche e della Rianimazione, IRCCS Fondazione Policlinico Universitario A. Gemelli, Rome, Italy

**Keywords:** Atypical haemolytic uremic syndrome, Pregnancy, Eculizumab

## Abstract

**Background:**

Atypical Haemolytic Uremic Syndrome is an acute life-threatening condition, characterized by the clinical triad of microangiopathic hemolytic anaemia, thrombocytopenia, kidney injury. Management of pregnants affected by Atypical Haemolytic Uremic Syndrome can be a serious concern for obstetric anesthesiologist in the delivery room and in the intensive care unit.

**Case presentation:**

A 35-year-old primigravida with a monochorionic diamniotic twin pregnancy, presented with an acute haemorrhage due to retained placenta after elective caesarean section and underwent surgical exploration. In the postoperative period, the patient progressively developed hypoxemic respiratory failure and, later on, anaemia, severe thrombocytopenia, and acute kidney injury. A timely diagnosis of Atypical Haemolytic Uremic Syndrome was made.

Non-invasive ventilation and high-flow nasal cannula oxygen therapy sessions were initially required. Hypertensive crisis and fluid overload were aggressively treated with a combination of beta and alpha adrenergic blockers (labetalol 0,3 mg/kg/h by continuous intravenous infusion for the first 24 hours, bisoprolol 2,5 mg twice daily for the first 48 hours, doxazosin 2 mg twice daily), central sympatholytics (methyldopa 250 mg twice daily for the first 72 hours, transdermal clonidine 5 mg by the third day), diuretics (furosemide 20 mg three times daily), calcium antagonists (amlodipine 5 mg twice daily).

Eculizumab 900 mg was administered via intravenous infusion once per week, attaining hematological and renal remissions. The patient also received several blood transfusion units and anti- meningococcal B, anti-pneumococcal, anti-haemophilus influenzae type B vaccination.

Her clinical condition progressively improved, and she was finally discharged from intensive care unit 5 days after admission.

**Conclusions:**

The clinical course of this report underlines how crucial it is for the obstetric anaesthesiologist to promptly identify Atypical Haemolytic Uremic Syndrome, since early initiation of eculizumab, together with supportive therapy, has a direct effect on patient outcome.

## Background

Haemolytic Uremic Syndrome (HUS) is an acute life-threatening condition, characterized by the clinical triad of microangiopathic haemolytic anaemia, thrombocytopenia, kidney injury [1, 2]. HUS belongs to the spectrum of thrombotic microangiopathy (TMA) syndromes, all defined by the coexistence of microangiopathic haemolytic anaemia, low platelet count and pathological evidence of endothelial cell damage [3]; in HUS, endothelial cell damage is mainly documented in the glomerular basement membrane, and renal failure is therefore one of the clinical hallmarks. By ‘atypical HUS’ (aHUS) we refer to all HUS cases not caused by the Shiga-toxin-producing Escherichia coli infection [4].

Pregnancy and its complications may precipitate a TMA in patients predisposed to complement dysregulation, entailing a higher risk of aHUS [5–7]. Pregnancy-aHUS is associated with high maternal morbidity and mortality, possibly leading to end stage renal disease and death. Eculizumab, a monoclonal antibody that targets complement protein C5, seems to effectively prevent organ damage and reduce mortality [8]. Nevertheless, there are few published reports regarding aHUS and treatment with eculizumab, due to the rarity of the disease and the rather recent approval of the monoclonal antibody. Therefore, management of patients with aHUS can be a serious concern for obstetric anaesthesiologist in the delivery room and in the intensive care unit (ICU).

Herein, we present the anaesthesiologic management of a primigravida, who presented with hypertension, haemorrhagic shock, and aHUS in the post-partum period and was then successfully managed and treated with eculizumab. Patient signed a consent, according to the protocol of the local Ethics Committee (Policlinico Universitario Agostino Gemelli Ethics Committee).

## Case presentation

A 35-year-old primigravida with a monochorionic diamniotic twin pregnancy presented in the operating room (OR) for an elective caesarean section at 36 weeks of gestation. The patient had a BMI of 26,6 Kg/m^2^ and a history of hypothyroidism, but her medical history and the pregnancy course were otherwise unremarkable (ASA class II).

On arrival at the operating room, she underwent a lower segment caesarean section under spinal anaesthesia performed with hyperbaric bupivacaine 0.5% 10 mg plus sufentanil 5 mcg and morphine 100 mcg intrathecally administered. The surgery was uneventful, without the need for vasopressors after neuraxial anaesthesia. Conversely, a tendence towards hypertension (mean arterial pressure persistently above 100 mmHg) was detected. Oxytocin 5 units in slow bolus and then 20 units by continuous infusion in 3 h were given to prevent uterine atony. Two healthy female babies were born (first baby’s Apgar scores of 7–9 and second baby’s Agar scores of 9–10 at 1 and 5 min).

Ninety minutes after the end of surgery, patient experienced fatigue and dyspnea in the Post-Anaesthesia Care Unit (PACU). She presented with an acute haemorrhage due to retained placenta and was urgently transferred to the OR for surgical exploration. Laboratory tests were preoperatively carried out and preoperative and postoperative tests are displayed in Table 1; they eventually confirmed only a reduction in hemoglobin level. Surgery was conducted under general anaesthesia with rapid sequence induction (Propofol 2 mg/kg and Rocuronium bromide 1,2 mg/kg) and maintenance with intravenous opioids (fentanyl 300 µg) and halogenated anaesthetics (Sevoflurane 1–1,5 MAC). Two large bore 14-gauge IV catheters were inserted; high-volume fluid resuscitation (ringer’s lactate solution 3000 mL + albumin 5% 500 mL), supplemental 20 units of oxytocin and 400 µg of rectal misoprostol were required. Due to the coexistence of preoperative anaemia and an estimated blood loss of 2500 mL, three units of red blood cells were given, together with fibrinogen concentrate (2 g) and tranexamic acid (1 g). A Bakri balloon was also inserted and maintained for the first 24 h after surgery. At the end of surgery, residual neuromuscular block was antagonized with sugammadex 2 mg/kg (160 mg).

**Table 1:** Laboratory tests during hospitalization

Day	Hb g/dL	PLTs *10^9^/L	WBC *10^9^/L	INR	aPTT sec	Fibr mg/dL	Creat mg/dL	BUN mg/dL	AST/ALT UI/L	LDH UI/L	Alb g/L	ATIII %	K^+^ mmol/L	Glu mg/dL	Others
1, pre- manual removal of placenta	7.1	174	12.7	1.02	30	257						66			
1, post-MRP	9	69	11.5	1.18	46.7	250	1	14				41		124	
1, PACU	6.7	40	17.2	1.14	43.8	260	1.4	18	76 / <5	1789		64	5.2	104	DD 28,628 ng/mL; TBil 1.8 mg/dL
1,ICU admission	8.2	39	17.1	1.12	45.5	271	1.6	20	n.d./ 27	2187	22	60	5.5	86	DD 21,098 ng/mL, TBil 1.5 mg/dL, Mg^++^ 1.3 mmol/L, direct Coombs test neg
2, ICU	6.9	29	14.5	1	47.2	360	2.1	21	105/40	2030	24	52	5.6	85	DD 7923 ng/mL, Sch neg, Apt < 7.75, ADAMTS13 Ag 424.8 ng/mL, ADAMTS13 Act 43.3%; B_2_ neg, aCL neg, LAC neg, TnI 1409
2, ICU	7.7	31	13.7	0.9	42.3	395	2.3	25	99/45	2356	22	60	5.9	104	DD 4930 ng/mL, Apt < 7.7, TnI 943
3, ICU	6.8	33	15.3				3	34	56/27	1743	23		5.4	102	Sch POS, TnI 493
3, ICU	5.8	25	10.6												
3, ICU	7.1	23	11.4	0.95	37.8	410	3.4	38	n.d./15	1484	25	80	4.4	104	
4, ICU	6.9	27	8.5	0.93	35.5	393	3.9	46	33/11	1492	22	82	4.2	76	DD 3726 ng/mL, Sch neg, Apt < 7.7 mg/dL, Prot 0.34 g/L, C3c 82.5 mg/dL, C4 12.1 mg/dL, CH50 14.7 Ueq/mL
4, ICU	8.6	26	10	0.93	35.1	405	4	51	36/9	1690	22	97		90	DD 4823 ng/mL
5, ICU	7.9	24	9.5	0.99	36.1	360	4.3	58	26/ <5	1381	28	100		135	Sch neg, Apt < 7.7, C3c 95.2 mg/dL, C4 15.5 mg/dL, CH50 17.8 Ueq/mL
6	6.4	24	9.5				4.7	74	n.d./ <5	1669	28		4.1	114	
7	7.7	20	10.4				4.6	96	n.d./ <5	1730	26			99	
8	7.5	29	10.5				4.4	92	n.d./ <5	1312	26		4.2		
9	7.4	38	7.8				3.8	95	n.d./ <5	1281	26		3.2	118	Apt < 7.75 mg/dL
10	6.8	41	9.4												
11	9.1	56	10				2.5	70	n.d./ <5	1131	31		3.3	83	C3c 138 mg/dL, C4 22.9 mg/dL
12	8.9	67	9.8				1.9	47	n.d./ <5		29		3.1	78	Apt < 7.75 mg/dL
13	8.7	98	8.9				1.4	35	n.d./ <5	872	30		3	68	
14	9.2	132	8.6				1.4	27	n.d./ <5	726	30		3.5	67	
15	8.8	160	8				1.2	19	n.d./ <5	631	28		3.1	74	
42	12.5	213	8.4				0.79	30		195			3.3		Apt 121 mg/dL, C3c 127 mg/dL, C4 31.8 mg/dL

*DD *D-dimer level, *TBil *Total bilirubin, *Sch *Schistocytes, Apt Aptoglobin (n.v. 45–320 mg/dL), *ADAMTS13 Ag* ADAMTS 13 antigen (normal values 520–960 ng/mL), *ADAMTS13 Act *ADAMTS13 activity (normal value > 50%); B_2_ = IgG and IgM antibodies to β2 glycoprotein I, *aCL *IgG and IgM antibodies to cardiolipin, *LAC *Lupus anti-coagulant antibodies, *TnI *Troponin I High Sensitivity, *Prot *Proteinuria (n.v. <0.15 g/L), *C3c *C3 complement protein (n.v. 90–180 mg/dL), *C4 *C4 complement protein (n.v. 8–32 mg/dL), *CH50 *50% Haemolytic Complement Activity (n.v. <70 Ueq/mL)

After emergence from general anaesthesia, a prolonged postoperative observation in PACU was accomplished by the attending anaesthesiologist. Blood tests confirmed anaemia (Hb 6.7 g/dL) and revealed severe thrombocytopenia (PLTs 40 × 10^9/L), hypofibrinogenemia, acute kidney injury (AKI) (K-DIGO stage 1, serum creatinine 1.4 mg/dL), hyperkalemia and a slight increase in transaminase level. One additional red blood cell unit was given, together with fibrinogen (2 g). Within a few hours, the patient progressively developed hypoxemic respiratory failure. A chest radiograph documented bilateral perihilar patchy opacities, and a diagnosis of pulmonary oedema was made. Non-invasive ventilation (NIV) with a full-face mask was provided and the patient was transferred to ICU.

At ICU arrival, the patient appeared alert and oriented, still tachypneic, tachycardic and hypertensive (sinus rhythm heart rate: 112 bpm, invasive blood pressure: 148/97 mmHg). NIV and high-flow nasal cannula oxygen therapy (HFNC) sessions were initially required. Serial chest x-ray and lung ultrasound assessments both confirmed signs of interstitial fluid accumulation and pleural effusion, together with a right perihilar infiltrate, primarily suspected of alveolar oedema, albeit a pneumonia could not be definitely ruled out. Hypertensive crisis and fluid overload were aggressively treated with a combination of beta and alpha adrenergic blockers (labetalol 0,3 mg/kg/h by continuous intravenous infusion for the first 24 h, bisoprolol 2,5 mg twice daily for the first 48 h, doxazosin 2 mg twice daily), central sympatholytics (methyldopa 250 mg twice daily for the first 72 h, transdermal clonidine 5 mg by the third day), diuretics (furosemide 20 mg three times daily), calcium antagonists (amlodipine 5 mg twice daily). In addition, a short course of empirical beta-lactam therapy for community-acquired pneumonia was prescribed.

On the first day of ICU admission, the patient complained about blurred vision and visual disturbances. An ocular fundus examination revealed bilateral exudative retinal detachment, and a corticosteroid treatment was initiated (intravenous methylprednisolone 40 mg daily for three days).

Blood tests were sent, confirming anaemia, thrombocytopenia, AKI, and revealing an increase in LDH level, a reduction in C3 and haptoglobin level, and a normal value of C4 and ADAMTS13 (Table 1). Direct Coombs test and schistocyte count were also normal. Antiphospholipid antibodies (including lupus anticoagulant, IgG and IgM antibodies to cardiolipin and to β2 glycoprotein I) were not detected. A diagnosis of atypical Haemolytic Uremic Syndrome was made and Eculizumab 900 mg was administered via intravenous infusion. The patient also received anti-meningococcal B, anti-pneumococcal, anti-Haemophilus influenzae type B vaccination.

During the next few days, the patient’s respiratory condition gradually improved, so that after the first 36 h standard oxygen therapy was sufficient. She remained hypertensive despite a massive antihypertensive therapy, forced diuresis, and negative fluid balance. Due to the persistence of severe anaemia, she received several blood transfusion units. The patient spent 5 days in ICU before being transferred to the Department of Internal Medicine.

During the hospital stay, her clinical condition progressively improved and vital parameters and fundoscopic examination returned to normal. Prophylactic antibiotics against bacterial meningitis were continued up to two weeks after vaccination. A multigene panel (C3, CD46, CFB, CFH, CFI, DGKE, ADAMTS13) did not reveal pathogenic variants associated with genetic aHUS. The patient received Eculizumab 900 mg twice more (once per week IV administration), attaining hematological and renal remission. She was eventually discharged from hospital 16 days after admission.

## Discussion

We presented a case report of a pregnant with several risk factors for aHUS (primigravidas, multiple gestation, pre-eclampsia, retention of placental tissue, haemorragic shock), who developed many of the life-threatening complications associated with hypertensive disorders (hypertensive crisis, pulmonary oedema, retinal detachment) and of a-HUS (anaemia, thrombocytopenia, acute kidney injury).

Eculizumab has recently been recognized as a good therapeutic strategy for a-HUS, but clinicians should have a high index of suspicion, as a prompt diagnosis and a target treatment may enable a full recovery. We recommend considering a-HUS among possible competing diagnosis in patients with risk factors, who rapidly develop anaemia, thrombocytopenia and severe renal failure in the immediate post-partum period.

As atypical haemolytic uremic syndrome (aHUS) is a complement-mediated disorder [8], it is hardly surprising that 10–20% of all aHUS cases are pregnancy-associated, as pregnancy itself represents a complement amplifying condition. This immune etiology is also shared by pre-eclampsia, where the leading mechanism is suspected to be an excessive maternal inflammatory response to fetal trophoblasts, regarded as antigenic agents, leading to circulating immune complexes formation, classical complement pathway activation, vascular damage and inflammation [9]. As evidence of this conclusion, preeclampsia is more common in primigravidas, in oocyte donation pregnancies, in case of hyperplacentation (e.g., multiple gestations, molar pregnancy), and it frequently resolves with placental delivery.

Interestingly, to explain why aHUS mostly occurs in the post-partum period, it has been proposed that the placenta could equally be a protective factor against complement activation. Indeed, by overexpressing two membrane bound proteins (Decay Accelerating Factor and CD59), placenta may negatively control the complement alternative pathway. Anyway, after placental delivery, when this regulatory mechanism is lost, aHUS may be triggered [5].

The clinical course of this report underlines how crucial it is for the obstetric anaesthesiologist to promptly identify atypical haemolytic uremic syndrome, because it has a direct effect on patient outcome.

The patient described in this case report had major risk factors for aHUS: she was in the postpartum period of a twin pregnancy, complicated by severe hypertension, retention of placenta, haemorrhagic shock.

On the basis of the clinical presentation of this patient, three different diagnoses were possible: severe pre-eclampsia and haemorrhagic shock, thrombotic thrombocytopenic purpura (TTP), or aHUS. Key elements to the diagnosis were markedly abnormal laboratory findings: anaemia, thrombocytopenia, elevated serum lactate dehydrogenase, high creatinine level. The normal value of ADAMTS13 testing ruled out TTP. The intricate part was to differentiate the pregnancy complications themselves from aHUS, with which they frequently co-occur, since post-partum haemorrhage and pre-eclampsia may share features of TMA. Eventually, the persisting anaemia, the very low platelet count level together with the normality of the other coagulation tests, the severe renal failure, all worsening in the immediate postpartum period, were consistent with aHUS. Even if we could not confirm the microangiopathic nature of anaemia until the third day in ICU, when peripheral smear finally confirmed the presence of schistocytes, the elevated LDH level and the decline in haptoglobin levels strongly suggested it from the beginning. Lastly, since the transaminase level was only slightly increased, HELLP syndrome was unlikely [2].

The clinical course of this case of aHUS once again underlines how necessary it is to differentiate among TMA syndromes, since a prompt medical treatment, together with supportive therapy, may improve the outcome. The absence of genetical variants does not preclude the diagnosis, since predisposing genetical variants may be identifiable in only 60% of all aHUS cases [4, 5], and should not delay therapy, since prompt initiation of eculizumab therapy seems to account for better outcomes [10]. Likewise, complement protein levels are normal in the 60% of patients with aHUS [11]. Anyway, the patient of this report presented with a slight decrease in C3 level and a normal C4 level, suggesting alternative pathway–mediated complement activation.

In a recent systematic review [12], the authors analyzed 60 cases of pregnancy-associated aHUS, revealing that the condition more frequently occurred in the post-partum period, after caesarean delivery, in nulliparous women, with mean delivery gestational age 36.4 weeks. Moreover, the diagnosis was often preceded by a pregnancy complication, as hypertension or pre-eclampsia (57%) and maternal haemorrhage (22%). Treatment with eculizumab favored remission (treatment with eculizumab 88% vs. no treatment with eculizumab 57%, *p* = 0.02) and was never associated with a composite outcome of persistent renal failure, dialysis or death (no case in the 17 patients treated with eculizumab, versus 9/37 patients not treated with eculizumab).

Besides, in accordance with the analysis of the observational non-interventional Global aHUS Registry, the risk of progression to end stage renal disease appears significantly higher for women with pregnancy-aHUS not treated with eculizumab (adjusted Hazard Ratio 0.08 (95% CI 0.01, 0.65; *p* = 0.019)) [13].

Due to the favorable side-effect profile and its potential efficacy, eculizumab should be considered the first-line treatment, although it is impossible to conduct trials on the issue because of the infrequency of aHUS, with an estimated incidence of 1 every 25,000 pregnancies [8, 14]. Patients treated with eculizumab must be given meningococcal prophylaxis, as meningococcal meningitis represents the major risk of terminal complement blockade [15–18]. When Eculizumab is not available, or in low-income countries, plasmapheresis should be offered and considered the best therapeutic option, although its outcome seems unsatisfactory [19–21].

## Data Availability

The datasets and clinical data used and/or reported in the current study are available from the corresponding author on reasonable request.

## Abbreviations

HUS: Haemolytic Uremic Syndrome
aHUS: atypical Haemolytic Uremic Syndrome
TMA: Thrombotic MicroAngiopathy
ICU: Intensive Care Unit
ASA: American Society of Anesthesiologists Physical Status Classification System
OR: Operating Room
PACU: Post-Anaesthesia Care Unit
AKI: Acute Kidney Injury
NIV: Non-Invasive Ventilation
HFNC: High-Flow Nasal Cannula
TTP: Thrombotic Thrombocytopenic Purpura
